# Integration of IoT in Small-Scale Aquaponics to Enhance Efficiency and Profitability: A Systematic Review

**DOI:** 10.3390/ani14172555

**Published:** 2024-09-02

**Authors:** Muhammad Aiman Hakim bin Zamnuri, Shuting Qiu, Muhammad Akmal Arif bin Rizalmy, Weiyi He, Sumiani Yusoff, Kakaskasen Andreas Roeroe, Jianguo Du, Kar-Hoe Loh

**Affiliations:** 1 Institute of Ocean and Earth Science, Universiti Malaya, Kuala Lumpur 50603, Malaysia; 2 Key Laboratory of Marine Ecological Conservation and Restoration, Third Institute of Oceanography, Ministry of Natural Resources, Xiamen 361005, China; 3 APEC Marine Sustainable Development Center, Xiamen 361005, China; 4 Faculty of Fisheries and Marine Sciences, Sam Ratulangi University, Manado 95115, Indonesia; 5 Faculty of Marine Biology, Xiamen Ocean Vocational College, Xiamen 361100, China

**Keywords:** Internet of Things (IoT) technology, operational costs, resource utilization, small-scale aquaponics, water quality parameters

## Abstract

**Simple Summary:**

The Internet of Things (IoT) can improve small-scale aquaponics, a sustainable farming method that combines fish farming with plant growing in water without soil, by making the process more efficient and profitable by optimizing resource use, closely monitoring water quality, and ensuring the best conditions for both fish and plants to thrive. Aquaponics is beneficial for the environment and can help ensure a steady food supply but presents challenges for small-scale farmers due to a lack of expertise in water chemistry and system upkeep, as well as high operational costs. Identified challenges in aquaponics operation include high water and energy costs, maintaining the right balance of fish and plants, and the risk of mosquitoes breeding in the water. This systematic review offers a comprehensive guide to setting up and maintaining an aquaponics system, including choosing the right fish and plants, designing the system, monitoring water quality, and feeding the fish. The importance of knowledge sharing among farmers is also highlighted to improve aquaponics practices. The integration of IoT into these systems can reduce the need for manual work and improve the availability of information related to system control, which could facilitate further adoption and optimization of aquaponics farming practices.

**Abstract:**

Aquaponics combines aquaculture and hydroponics to offer a sustainable approach to agriculture, addressing food security issues with minimal environmental harm. However, small-scale practitioners face challenges due to a lack of professional knowledge in water chemistry and system maintenance. Economic hurdles, such as operational costs and energy-intensive components, hinder the viability of small-scale aquaponics. Selecting suitable fish and plant species, along with appropriate stocking densities, is crucial. Media Bed (MB), Deep Water Culture (DWC), and the Nutrient Film Technique (NFT) are commonly used hydroponic techniques. This study outlines optimal conditions, including water quality, temperature, pH, and nutrient concentrations, essential for symbiotic fish and plant cultivation. Integrating IoT technology enhances efficiency and profitability by optimizing resource utilization, monitoring water quality, and ensuring optimal growth conditions. Knowledge sharing among practitioners fosters innovation and sustainability through collaborative learning and best practices exchange. Establishing a community for knowledge sharing is vital for continuous improvement, advancing small-scale aquaponics towards a more efficient and sustainable future.

## 1. Introduction

One problem currently facing all of humanity is the food demand can no longer be maintained by additional natural resources and land exploitation [1]. Hence, it is crucial to create a novel form of aquaculture that is eco-friendly and sustainable, such as aquaponics. In a functional aquaponics system, the fish, plants, and microorganisms all live together in a mutually beneficial relationship: the fish can serve as a source food for humans, and their waste could be a source of nutrients for the plants in the system [2]. As for microorganisms, they are important in the process of nitrogen fixation by plants in the system [3] and they can work together to reduce the accumulation of nitrogenous compounds, which are typically produced by aquaculture [4]. And it can be classified based on different purposes, including commercial, educational, recreational, research, or family self-consumption [5]. Due to the characteristic of being able to adapt to urban environments (such as the backyard or balcony), small-scale aquaponic systems for family use have seen a surge in usage around the world [6].

**Figure 1:**
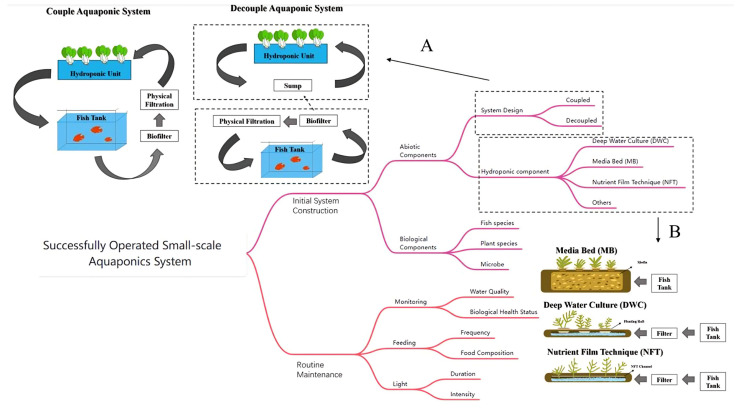
Flowchart of a small aquaponics system. (**A**) Differences between coupled and decoupled aquaponic system. (**B**) Three main types of hydroponic components used in aquaponics: MB, DWC, and NFT.

According to the Food and Agricultural Organization [7], a small-scale aquaponics system is defined as one that consists of a fish tank with a volume of approximately 1000 L and an area for plant cultivation of around 3 m^3^. A successful small-scale aquaponics system consists of two main parts: the initial system construction and routine maintenance, which are both crucial [8]. The first part is further divided into the biotic and abiotic components. The abiotic components primarily involve system design and hydroponic nutrients. There are two categories of aquaponic systems depending on their design: (1) coupled and (2) decoupled [9]. Aquaponics systems nowadays (Figure 1) are primarily categorized based on their hydroponic structure and whether they involve a closed water cycle or not, with common forms including Media Bed (MB), Deep Water Culture (DWC), and the Nutrient Film Technique (NFT), and all other forms are referred to as others [10]. As for the biotic components, they refer to the fish and plant microbiota. The selection of fish, plants, and microorganisms and the ratio need to be considered comprehensively based on their suitability for the water quality and the symbiotic nature of the entire system [11]. Routine maintenance involves monitoring, feeding, and lighting. Controlling the parameters and factors (such as light, temperature, pH, moisture, etc.) in a greenhouse and symbiotic environment can be very challenging [1], especially when participants are initially attempting without possessing relevant knowledge or having received related training. To make sure the system is in a good condition, it requires not only expertise or mature experience to make judgments but also a large number of labor work to monitor the system.

With the implementation of automation, smart strategies, and IoT in aquaponics systems, it appears that aquaponics has entered a new era [1]. What is more, now there are many successful cases of operation; various parameter sensors can be implemented to collect real-time data, which is then uploaded to a cloud database. In general, IoT consists of three layers which are the Perception Layer that is used for sensing, Network Layer for data transmission, and Application Layer for data storage and manipulation [12]. The automatic adjustment of parameters within the optimal range, such as temperature control, light control, humidity maintenance, water level control, and automatic feeding, can be achieved based on predetermined settings. In addition, users can receive prompts or alerts on their smartphones and make necessary adjustments to the system [13,14], including low-cost ones [15]. Furthermore, based on computer vision technology using deep learning algorithms, the analysis of fish movement behavior and assessment of their health can be achieved through cameras. This enables the determination of the optimal stocking density and water quality [16]. To sum up, the benefits of intelligent automation are significant reductions in manual work and tighter control over the system [1]. Users can make data-driven decisions rather than relying on experience, which is very friendly for novice players who do not have relevant knowledge reserves or have not received additional training.

With the premise that self-governance in households has been evaluated as one of the main social motivators for a small-scale aquaponics system [5], it is worth considering how to effectively integrate the emerging automation and IoT technologies with small-scale aquaponics to enhance productivity and address costs. Although the use of intelligent monitoring and IoT in aquaponics has been reviewed, the focus has been on large-scale, commercial aquaponics systems [1]. To the best of our knowledge, the design and application of automation and IoT in small-scale aquaponics systems have been explored by many researchers from different countries for various purposes. However, a comprehensive summary of these papers has not yet been compiled, leaving gaps in our understanding of this technology. Therefore, in this article, we systematically reviewed the problems, challenges, and opportunities encountered in the development process of aquaponics systems, and then based on the characteristics of small-scale aquaponics systems, we aimed to determine the following: (1) the main challenges that small-scale aquaponics systems face; (2) the technically and economically feasible suggestions on both the initial construction and later daily maintenance of the system in terms of economy and efficiency; (3) the solution of how to integrate the Internet of Things and automation into small-scale aquaponics systems to reduce labor and time costs while increasing productivity; (4) a vision that combines automated, intelligent home self-contained hydroponics. The information provided by this study is significant for those who are interested in small-scale aquaponics technology worldwide. It can help people to establish aquaponics systems at home within an affordable cost, providing a sustainable, safe, and healthy source of food. It is hoped that the results of this study can provide a reference answer to the sustainable development of food production and meet the growing demand for food security under the context of the imminent problem of food shortage.

Small-scale aquaponics systems are typically compact, suitable for home or backyard use. They integrate fish farming with soilless plant cultivation in a closed-loop system. These setups are often low-cost and require minimal space. Compared to commercial aq-uaponics which requires more significant investment, space, and technical expertise, small-scale setups are easier to manage but produce less yield compared to commercial operations. The comparison between small-scale and commercial aquaponics is shown in Table 1.

## 2. Materials and Methods

### 2.1. Construction of Database

The database establishment of published research on aquaponics is achieved by using the topic search term (aquaponic* AND fish* AND (nitrogen OR ‘vegetable waste’ OR ‘fruit waste’ OR ‘nutrient resources’ OR sustainability OR ‘urban farming’ OR ‘RAS’ OR ‘small-scale’)) in the Web of Science (WoS) on 8 March 2023. A total of 442 publications were obtained from a range of document types including articles, review papers, academic conferences, and others. The earliest publication identified within the search parameters was in 2007.

In order to facilitate a more comprehensive and rigorous review, Scopus-indexed databases were utilized with TITLE-ABS-KEY (aquaponic* AND fish* AND (nitrogen OR ‘vegetable waste’ OR ‘fruit waste’ OR ‘nutrient resources’ OR sustainability OR ‘urban farming’ OR ‘RAS’ OR ‘small-scale’)), resulting in a total of 327 publications retrieved on 10 March 2023.

**Figure 2:**
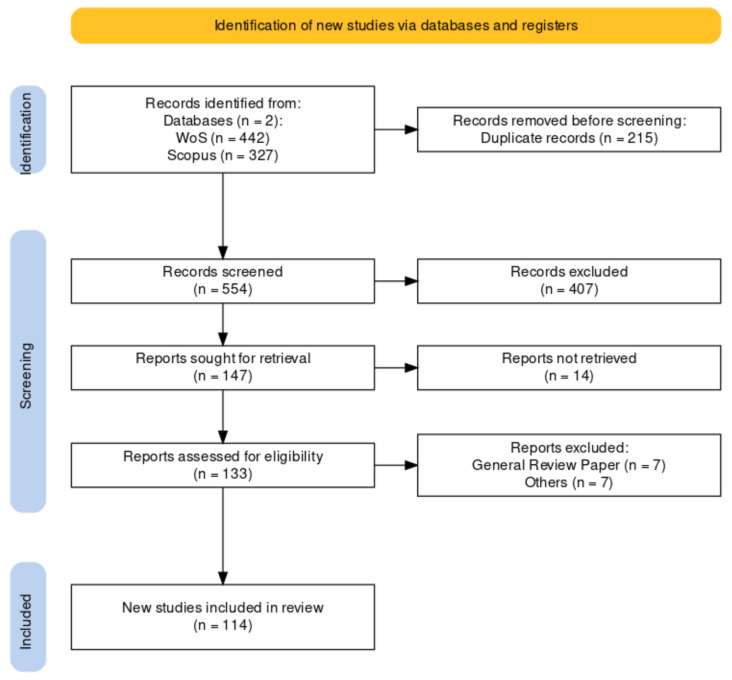
The PRISMA flow diagram was created based on searches conducted on the Web of Science (WoS) and Scopus databases.

A sum of 769 papers that were published was identified after merging the findings from both databases. Each paper’s title, abstract, year of publication, authors, and digital object identifier (DOI) were reviewed manually using R 4.2.1 [17] and the metagear package [18]. The preferred reporting items for the systematic and meta-analyses (PRISMA) flow chart were used to construct a more comprehensive and ideal meta-analysis, utilizing the R PRISMA2020 flow diagram package [19], as depicted in Figure 2.

All the literature screening was conducted manually and, for better organization, was divided into four categories: (1) the existing literature reviews; (2) studies related to participants in aquaponics; (3) experimental studies on various parameters of aquaponics; (4) the application of the Internet of Things in aquaponics.

We reviewed a total of 114 articles related to small-scale aquaponics, with most of the articles focused on the USA, China, and Europe, followed by Africa. And according to Hao et al. [20], in the field of aquaponics research, the United States stood out as the most innovative country, publishing more papers than any other country in the world. And each country has their unique research focus. For example, many studies in China were conducted on the application of molecular biological methods and Internet of Things (IoT) technology. At the same time, European researchers, particularly those in the Netherlands, Germany, Italy, and Switzerland, explored the multi-faceted nature of aquaponics, including in public administration and government law as well as its contribution to urban studies and biodiversity conservation.

### 2.2. Trends in Academic Research

**Figure 3:**
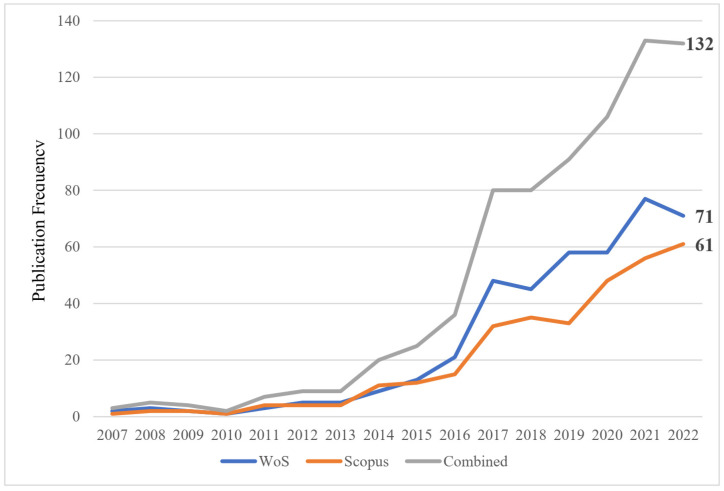
Trends and publication numbers of articles related to aquaponics in the two main databases.

In this review, we have compiled a total of 742 articles published between 2007 and 2022. Please note that 27 articles published in 2023 were not included as the review was conducted in early 2023 and the data for that year were incomplete. Our analysis revealed a positive trend in the field of aquaponics, which is illustrated in Figure 3. The combined database of WoS and Scopus publications was sorted by year, and an interesting observation was made. There was a sudden increase in the number of aquaponics studies in early 2016, possibly due to the increased attention to food safety and sustainable development. In 2019, the COVID-19 outbreak prompted more people to take an interest in aquaponics as a means of supporting themselves and reducing their risk of exposure to infection in public places. Another reason for the sharp increase in the number of studies in 2019 may be that in 2018, over 113 million people in 53 countries suffered severe hunger and required urgent assistance in food, nutrition, and livelihood (IPC/CH Phase 3 or above) [21], making it imperative for all of humanity to find a solution to this problem.

## 3. Results

### 3.1. Obstacles in Sustainable Aquaponic Farming

#### 3.1.1. Lack of Professional Knowledge among Practitioners

While interest in small-scale aquaponics has expanded exponentially, most practitioners enter as backyard hobbyists lacking the technical experience needed for commercial implementation. Love et al. [22] determined 80% of small aquaponics operators surveyed felt inadequately informed to make appropriate technology choices for their systems. Practical knowledge limitations exist around the nitrification process that allows aquaponics to function, including managing biofilters to facilitate complete ammonia conversion [23]. Gaps also persist around maintaining optimal water quality parameters [24], preventing disease outbreaks in the intensive production environment [25], and balancing the nutritional needs between plants and fish [26]. Many small farmers struggle with the level of precision and calibration required to nurture sensitive aquatic ecosystems. Aquaponics also integrates multiple disciplines like aquaculture, hydroponics, system maintenance, and more for which targeted skills need further development [9]. While emerging technologies like remote sensors have eased system monitoring, the availability of region-specific training programs and distributed learning networks remains unequal [27].

Understanding and managing the water chemistry, biofilter nitrification, and the dynamics of the nutrient film in hydroponic systems are crucial skills for enhancing productivity and increasing profit margins. For small-scale businesses, lack of such expertise given focused training requirements can negatively impact plant yields or cause catastrophic fish losses leading to poor economic viability [28]. Understanding the fundamentals of the nitrogen cycle facilitates strategic supplementation avoiding detrimental fluctuations. Practical skills enable the mastery of components that are specifically designed to perform essential functions, such as removing solids. Knowledge around the prevention and management of common diseases also optimizes fishes and plants’ health in intensive recirculating systems [29]. Market fluency regarding high-value crops, distribution channels, and options for value-added products allows strategic business modeling [30].

#### 3.1.2. Economic Challenges

Small-scale aquaponics operations face a variety of financial difficulties, such as careful consideration of operational costs, input–output relationships, and market dynamics. One of the key challenges lies in balancing the costs of water, energy, and fish feed. Love et al. [22] found that small-scale aquaponics systems experience about 1% daily water loss, necessitating an annual average of 35,950 L for replenishment, potentially posing cost challenges, but suggest mitigating measures such as efficient filtration and recirculation to reduce water usage.

Energy for heating, pumping, and lighting also contributes heavily to operational expenses. Parajuli et al. [31] explained that creating the ideal conditions for plant and fish growth in the controlled environment of aquaponics includes maintaining water quality, temperature, humidity, light, and pH, and requires the use of energy-intensive components, resulting in a higher demand for energy. Optimizing energy efficiency should be prioritized. Learning from recirculating aquaculture systems (RAS), it is important to note that ventilation and water-cooling play crucial roles in electricity consumption.

Feed for raising fish makes up over 50% of variable production costs for most small aquaponics businesses [32]. Determining the appropriate amount of feed is crucial. The practitioners are facing difficulty with feeding intervals, as too frequent feeding can cause unnecessary stress, while infrequent feeding can negatively impact the fish’s nutritional needs. Excess feeding also led to the accumulation of uneaten food, which can lead to water quality issues [33]. While formulas geared for aquaponics exist, cost analysis studies advise buying commercial feed based only on fish nutritional needs. This allows balancing feed conversion ratios and growth rates to save on feed expenses. Knowing exact feed consumption and fish biomass production metrics through meticulous monitoring enables fine-tuning of feeding regimes for cost control.

The economic sustainability of small-scale aquaponics heavily relies on the optimization of production efficiency. Targeting local premium niche markets for chemical-free, sustainably grown produce and utilizing direct marketing channels like community-supported agriculture programs can prioritize revenue [34]. Diversifying the systems by incorporating high-value aquatic plants, ornamental fish, shrimp, and duckweed can supplement income through a varied product portfolio [35]. Seeking collaborations with urban farmers’ affiliations and networks can open up marketing, funding, and educational opportunities to boost profitability [36]. Since energy accounts for nearly 30% of variable costs, renewable technologies like solar, the use of energy-efficient pumps, and leveraging natural light drastically cut expenditures [37]. The government should provide grants and loans, focusing on aquaponics that offer a different way to get money, making it easier for people to access capital for their farming projects.

#### 3.1.3. Mosquito Breeding Ground

Aquaponics systems provide standing water bodies, which serve as ideal breeding grounds for mosquitoes. These insects lay their eggs in stagnant or slow-moving water, and the warm temperatures in tropical and subtropical regions accelerate their life cycle [38]. The presence of mosquitoes such as Anopheles and Culex may compromise fish health and productivity by transmitting pathogens or parasites [39]. Proper water management, including circulation and aeration, is essential to prevent mosquito breeding in aquaponics systems [40]. Introducing predators like guppies and using larvicidal bacteria such as Bacillus thuringiensis israelensis (BTI) can effectively control mosquito populations [41,42] and avoid the spread of mosquito-borne diseases [38]. Additionally, installing screens or covers over water bodies helps restrict mosquito access while maintaining air circulation and light penetration [43].

The Mosquito Ovitraps IoT Sensing System developed by Aldosery et al. [44] can help monitor and provide a solution for mosquito breeding in aquaponics systems by using IoT sensors together with weather and water quality sensors. These sensors monitor parameters, including temperature, humidity, pH, and dissolved oxygen, that are responsible for the surge in mosquitoes. The information is forwarded in real-time to a cloud server where practitioners can monitor their systems remotely to identify conditions that may encourage mosquito breeding. This would allow for effective targeting of mosquito control measures.

### 3.2. Information on the Maintainance and Management of Aquaponics Systems

#### 3.2.1. Selecting Fish, Plant Species, and Stocking Density

For a successful aquaponics system, careful consideration must be given to the selection of fish and plant species, along with determining the appropriate stocking density. Nile tilapia, known for its abundant byproducts, including skin, bones, fins, heads, guts, and scales, has become a highly favorable choice for aquaponics. Nile tilapia has emerged as a crucial collagen source, with its skin providing high yields of valuable acid or pepsin-soluble collagen [45]. Being among one of the largest farmed fish globally, tilapia is fast-growing, adaptable, and easy to process into fillets [46]. In addition, the catfish is also a popular choice for aquaponics. The catfish thrives in temperate to subtropical climates, making it a suitable aquaculture species across regions of the Americas. With high reproduction rates, resilience to handling stress, and rapid growth, catfish shows promise for controlled breeding and juvenile production to supply markets [47]. Besides tilapia and catfish, largemouth bass, crappies, rainbow trout, carp, and Koi are also a recognizable choice among farmers. The selection of these species is based on factors like market value, nutritional quality for consumption, and the volume produced in conventional systems.

For plants, high-value vegetables such as basil, herbs, tomatoes, lettuce, salad greens, chard, pepper, kale, and cucumbers are excellent options [24]. These plants are often grown alongside fish in closed-loop systems where fish waste serves as a nutrient source for the plants. Different plants in aquaponics systems prompt distinct nitrogen transformations due to their varied growth characteristics and nitrogen utilization capacities [48]. Plants with larger root surface areas play a crucial role in fostering the growth of nitrifying bacteria by providing ample surface area for the development of the exopolymeric substances (EPS) that shield them [49].

According to Rakocy [50], the fish to plant ratio is based on the feeding rate ratio. The feeding rate ratio in aquaponics refers to the amount of fish feed provided per square meter of the plant growing area, measured daily. Taking the raft hydroponic system as an example, the recommended feeding rate ratio ranges from 60 to 100 g/m^2^/day [50]. This means that for every square meter of plant area, between 60 and 100 g of fish feed should be provided daily to maintain a balanced ecosystem. Generally, the recommended fish to plant ratio of one pound of fish biomass to every three to five square feet of plant growing area in an aquaponics system serves as a fundamental guideline for establishing and maintaining a balanced ecosystem [51]. Fish biomass, representing the total weight of fish within the system, contributes to ecosystem dynamics by consuming algae and organic matter, thus providing essential nutrients for plant growth [52]. Conversely, the plant growing area denotes the space designated for plant cultivation, where plants play a crucial role in converting fish waste into usable nutrients for both fish and plants [53].

#### 3.2.2. System Construction

The design of an aquaponics system requires careful planning to create an optimal environment for raising fish and plants symbiotically. Central to the setup are the fish tanks, containing varieties of fish such as tilapia, trout, catfish, or Koi. Love et al. [22] established four fish tank systems as one experimental unit. Multiple fish tanks allow raising and harvesting of fish at varying developmental stages in a staggered fashion [54]. For small-scale aquaponics systems, a water tank volume ranging from 50 to 200 L is recommended. A 50 L tank can accommodate between 50 and 100 small fish, while a larger 200 L tank can support a higher stocking density [55]. However, it is important to note that a higher stocking density may result in slower individual fish growth due to increased competition for resources and the accumulation of waste products. Conversely, the elevated waste output from a denser fish population can accelerate plant growth by providing more readily available nutrients for the plants. Alongside the fish tank in the RAS subsystem, there were also filter tanks containing mechanical and biological filtration to remove solids and convert nitrogenous wastes, mechanical and biological filters, and plant tanks containing the hydroponic growing systems [56], such as Media Bed (MB), Deep Water Culture (DWC), and the Nutrient Film Technique (NFT). Vertical gardens and living wall components design are important to optimize nutrient delivery and water efficiency by utilizing various hydroponic techniques such as DWC, the NFT, ebb and flow, or growing towers with aeroponics [57]. These systems are adaptable for a small scale, especially in urban environments characterized by small fish tank volumes, extensive stocking densities, and small plant areas.

A study by Filep et al. [58] depicted a system that, although smaller in scale, still fell within the small-scale threshold of aquaponics set by the FAO. Their system consists of a fish tank sized to hold a 450 L water volume; here, their system does not reach close to the FAO figure and is less than half. The growing space for plants was 1.06 m^2^ within their system and 0.37 m^3^ in volume, and, like the others, used a media bed full of river gravel. This plant growing space is significantly less than the recommended FAO’s 3 m^3^. This system had some key components common in small-scale aquaponics, for instance, the following: an adjustable water pump rated at a flow rate of 150 to 1000 L/h, a bell siphon giving an effective irrigation of the media bed, an aerator for water oxygenation, and a horticultural lamp supporting photosynthesis in plants. Another study by Menon et al. [59] demonstrated that even a setup with only a 55 L fish tank and an area of 0.11 m^3^ media bed volume could support the plants of chili, ginger, and onion along with growing tilapia. This proves that even smaller setups than those previously discussed have demonstrated success in small-scale aquaponics.

In aquaponics system design, the power supply is a critical component, especially for urban environments where space and energy efficiency are crucial. The power requirements can vary significantly based on factors like the system size, number of fish tanks, filtration methods, and hydroponic growing techniques. For small-scale urban aquaponics systems, it is essential to consider energy-efficient solutions including the use of solar panels to power the system, especially in regions with abundant sunlight. According to Dbouk and Khalife [60], incorporating a solar power in aquaponics systems can achieve a 60% reduction in the required space for planting. This reduction in space not only saves physical resources but also the energy needed to maintain and operate the system. With less space, the system becomes more compact and efficient, leading to a 30% increase in productivity. Integrating energy-saving devices such as LEDs lighting and efficient pumps also can optimize energy usage in an aquaponics system. LEDs has lower power consumption, a longer lifespan, and reduces heat emission [61]. Additionally, choosing the right pump is crucial to lower energy consumption. Using a high-powered pump in aquaponics systems may result in increased energy consumption, higher risk of mechanical failure, and more expensive maintenance compared to energy-efficient pumps [62]. Overall, careful planning and the integration of energy-efficient solutions, such as solar power, LED lighting, and high-efficiency pumps, can significantly improve the system’s overall productivity and sustainability.

The integration of IoT in aquaponics streamlines system monitoring and control, enabling real-time data collection, automated adjustments, and remote management through a centralized dashboard. A Raspberry Pi equipped with a WiFi dongle serves as a central hub for data processing and communication in such a system. This makes it possible to communicate with cloud services smoothly and create an intuitive IoT dashboard. Important sensors are installed throughout the system in critical locations throughout the building process. To continuously monitor the temperature of a water tank, for example, a DS18B20 waterproof temperature sensor can be fitted [63]. This sensor provides vital information for maintaining ideal conditions for fish and plants. A water level sensor, which notifies the Raspberry Pi to cease pumping water when the water reaches a specific level, might be included to avoid overflow problems. In order to operate motors and lights, the design can also include smart outlet systems with relays and related drive circuits [63]. This configuration adds a level of convenience and control by enabling users to remotely toggle these components on or off via the dashboard. This IoT-integrated aquaponics system is inexpensive and straightforward, yet it offers consumers useful features like real-time temperature measurements and system alarms.

#### 3.2.3. Water Quality Monitoring and Control

Water quality is a fundamental aspect of aquaponics, as it directly impacts the health and productivity of both the fish and the plants. The regular monitoring of parameters such as pH, ammonia, nitrite, nitrate, and dissolved oxygen is essential to prevent water quality issues that can lead to disease and death in fish, as well as stunted plant growth [64]. Automated monitoring systems and water treatment technologies can help maintain water quality within the desired parameters [33]. Utilizing IoT for monitoring water quality in aquaponics offers the advantages of saving time and enabling remote control from anywhere, preventing potential harm to fish and plants [65]. Nutrient cycling also plays an important role in aquaponics by facilitating the efficient exchange of nutrients between the aquaculture and hydroponic components of the system. Fish waste, which is rich in ammonia, is converted into nitrites and then nitrates by nitrifying bacteria, enabling the nutrient uptake by plants [66]. At the same time, the absorption of nitrates and other dissolved nutrients by plants effectively removes them from the water and helps maintain good water quality for the fish [67]. By maintaining water quality and optimizing nutrient dynamics, aquaponics systems can achieve sustainable and productive food production.

#### 3.2.4. Fish Feed Selection and Plant Fertilization and Pest Control

The type of fish feed used is critical as it influences both the health of the fish and the level of nutrients available to the plants. Li et al. [68] recommend using extruded feeds, as they are more digestible and produce less waste compared to pellet feeds. The protein content of fish feeds is varied based on their metabolic activities. Herbivorous fish such as tilapia require lower protein (30–40%) [69] than omnivores like catfish (30–40%) [70] and carnivores like bass (41–46%) [71].

The plants rely on the nutrients provided by the fish waste, rather than traditional fertilizers. However, there may be instances where supplemental fertilization is necessary to address specific nutrient deficiencies. Romano et al. [72] suggest using organic, water-soluble fertilizers, such as Black Soldier fly frass, to provide additional nutrients without disrupting the system’s balance. It is important to monitor plant growth and nutrient levels carefully and make adjustments to the supplemental fertilization as needed. Maintaining a healthy and balanced aquaponics system can help prevent the proliferation of pests and diseases. Graber and Junge [73] recommend implementing integrated pest management strategies, such as the use of beneficial insects, physical barriers, and the application of organic, plant-based pesticides. Additionally, proper sanitation, good water quality, and the selection of pest-resistant plant varieties can help mitigate pest and disease issues in aquaponics systems.

### 3.3. Integration of IoT in Small-Scale Aquaponics

#### 3.3.1. Remote Monitoring and Control of Aquaponics Systems

IoT technology enables users to remotely monitor parameters through integrated sensors and connected devices that are centralized around a central hub or cloud-based platform [74]. The design comprises a microcontroller and various sensor modules for monitoring water quality, the plant environment, and growth conditions. It incorporates a microcontroller such as Raspberry Pi (manufactured by Sony UK Technology Centre in Pencoed, Wales, UK), NodeMCU (produced by Espressif Systems in Shanghai, China), and Arduino (available from arduino.cc in Italy) for image processing and data analysis with a Wi-Fi interface to get freely onto the internet [75]. Specific sensors monitor dissolved oxygen (DO), light intensity, and plant height, providing real-time data on these parameters. The gathered information from sensors is sent to the Blynk IoT platform (v1.45), an iOS and Android software that enables internet-based device control for the Arduino, Raspberry Pi, and NodeMCU. Subsequently, the information can be sent towards a local server and then transferred to a mobile device [12]. The system includes ESP8266 wireless communication modules for transmitting data to cloud servers, enabling remote monitoring and control of the aquaponics system [76]. Actuators are also an integral part of this setup, where they control a number of parameters and make adjustments that are needed in respect to changes in the environment. These actuators typically comprise relay switches that allow things to be turned on and off in a self-supporting manner without human input [74].

IoT integration in small-scale aquaponics reduces manual labor and provides robust process control through the increased accessibility and connectivity of parameters [1]. Automation has been shown to boost productivity, decrease human error, and save time and labor [1]. IoT-integrated systems allow owners or staff to receive real-time information via a semi-automatic microprocessor, sending alerts to connected personal computers. Smartphone applications streamline monitoring, saving time and reducing workload [77]. IoT algorithms analyze historical data and sensor readings to identify patterns and predict potential breakdowns [78], enabling proactive maintenance and uninterrupted operation.

The integration of the aquaculture and hydroponic system in one would have many parameters that would require frequent attention, but IoT technology has made much of the maintenance tasks considerably easier and automated. Farhan et al. [79] combined an Arduino Nano microcontroller and a Raspberry Pi 3 Model B+ (produced by Cytron Technologies in Penang, Malaysia) to automate some of the key elements in an aquaponics system. Through its three analog sensors, the system displays in real-time the measurement data of light intensity, pH, and water level on the LCD display. Further, with the help of relays, it controls a 12 V pump and LED lights. For remote management, the device is fitted with a portable Wi-Fi modem; hence, all the devices may be controlled via Telegram. The system is powered by a combination of a 250 V power supply and a step-down transformer, while the Raspberry Pi enables data flow and connectivity. They found that the ADC values drop progressively from 290 down to 0 as the level of water falls from 0 to 4 cm, and then to a dry state. The linearity in the decrease in the ADC value certainly proves that the sensor precisely measures the water level so that the automation system may act accordingly to start the pump if necessary.

#### 3.3.2. Key Parameters and Sensors

There are several key parameters that require continuous monitoring so that optimal growth conditions are maintained. The ideal range of these parameters such as temperature, pH, dissolved oxygen, and nutrient levels are very specific to fish and plant species, as well as the system’s design. Available sensors provide an accurate, real-time measurement for these parameters to ensure that they stay within the recommended ranges for the optimum growth and prevention of system failures. The breakdown of each parameter with respect to its optimal ranges and the corresponding sensors is shown in Appendix A Table A1.

## 4. Discussion

### 4.1. IoT Solutions for Optimizing Aquaponics Systems

#### 4.1.1. Parameter Optimization

The adoption of IoT in aquaponics systems allows gathering and analyzing several metrics or measurements that are vital to the productivity and well-being of plants and fish. These characteristics include salinity levels, pH, dissolved oxygen (DO), and water temperature, among others. Mahmoud et al. [80] designed an automated equipment that can analyze these measures and determine whether adjustments are needed to maintain a perfect environment for fish and plant growth. These comparisons are made against established ideal values. When the system detects a deviation from the ideal parameters, it transmits the information to the central control unit, which instructs the heaters, air pumps, valves, and water pumps among other aquaponics system components to adjust as needed. For instance, if the water temperature drops below the optimal threshold, the system would automatically activate the heater to raise the temperature back to the optimized level that suits the system’s needs. Therefore, IoT technology automates the management of crucial parameters and reacts proactively to changes in the environment.

#### 4.1.2. Nutrient Optimization

A compelling example of how IoT technology optimizes nutrient use is demonstrated by Dhal et al. [81], who created an advanced nutrient regulation model by combining IoT and machine learning. Their system consists of three main subsystems: a sensor system for measuring the calcium and ammonium levels connected to a Raspberry Pi, a Python-based feedback loop for continuous monitoring and adjustment, and an actuator subsystem for precise nutrient dispensing. Through the use of an advanced feature selection pipeline, they determined that calcium and ammonium were the most important nutrients to keep an eye on. When levels drop below the ideal average, the system automatically dispenses nutrients that have been saved. It monitors these nutrients continuously. This degree of accuracy and automation minimizes the loss of costly nutrients and lowers the possibility of overfertilization, which can be harmful to fish as well as plants. Additionally, the system may raise production yields by continuously maintaining optimal nutrient levels. This IoT-driven strategy enables practitioners to rely on automated, data-driven nutrient regulation, which is especially advantageous for plants that are sensitive to an overabundance of nutrients.

#### 4.1.3. Feeding Optimization

Abu-Khadrah et al. [82] designed an automated fish feeder using IoT for small-scale aquaponics systems. The central control unit for the system was a Raspberry Pi, accompanied by a Pi Camera for live video streaming that enabled the remote monitoring of fish and feeding behavior. A stepper motor, powered by a Python script on the Raspberry Pi, rotates a feed container with strategically placed holes to dispense fish feed. The feeder is capable of being powered from a 5 V supply and operates in two modes: automatic, which dispenses feed at pre-set intervals, and manual, allowing remote control for feeding the fish via a web interface. The system’s reliance on the standard 5 V power supply may pose challenges for long-term, off-grid operation. In this context, a solar-powered energy solution will have to be sought out to ensure the sustainability of the system. Silalahi et al. [83] have demonstrated an efficient integration of solar electricity to power IoT-based aquaponics systems. Their system included a 50 Ah battery and a solar panel, which provided a reliable and renewable energy supply. Their model had a buffer mechanism that would help as a holder and regulator in terms of the orientation and angle for the solar panel to maximize performance. Additionally, combining the technologies of Artificial Intelligence (AI) with IoT sensors of the automated fish feeder can enhance the precision in the feeder and the decision-making process. Real-time analysis of feeding behavior of fish is achievable through the use of the camera feed that is analyzed by AI algorithms able to observe and translate the fish behavior. This makes it possible for the system to be able to accurately measure the amount of food fed to the fish without wasting any or overfeeding them [84].

#### 4.1.4. Energy Optimization

When compared to traditional farming methods, aquaponics systems have shown to be more water efficient. Typically, water loss in aquaponics systems ranges from 0.3% to 5%, mostly owing to plant transpiration. In traditional farming, water loss was observed to be 10% [85]. Incorporating IoT technology further improves water efficiency, as shown by Sunardi et al. [86]. With a filtration stage to guarantee water quality, their system uses a water pump to circulate water using a nutrient film approach from the fish tank to the plants. The use of IoT technology, particularly a turbidity sensor, makes it possible to monitor water impurities that can be dangerous to fish in real-time. This sensor triggers an alarm, telling users to control the water pump as necessary to ensure the best possible water quality. Their system is also equipped with a light sensor that detects the absence of solar energy, enabling users to turn on installed lighting in order to help with plant photosynthesis when the intensity of the sunlight is inadequate. This feature aids in attaining maximum plant growth without expending extra energy on lighting when there is sufficient sun intensity.

### 4.2. Establishing a Community for Knowledge Sharing

Small-scale aquaponics practitioners often begin with limited prior knowledge or training [87]. Sharing experiences and insights enhances understanding and provides valuable information on system design, nutrient management, and maintenance [88]. Access to information enables practitioners to make informed decisions and optimize operations [89], while collaboration stimulates innovation and creativity [90]. Exchanging ideas, experiences, and experimental results explores new approaches and technologies for improved efficiency, productivity, and sustainability [91,92]. Knowledge sharing contributes to sustained industry success and growth by equipping practitioners with necessary skills and support systems [27]. Adopting good practices increases the reputation and viability of aquaponics as a sustainable farming method [93], attracting more investors, consumers, and stakeholders [94].

Aquaponics combines hydroponics and aquaculture in a cycle of waste repurposing, with biotic and abiotic factors established for system efficiency [95]. Without the controlled factors, the system will be equivalent to that of any typical aquatic habitat that has both the factors, however, with each component to not be specific or of the precise proportion and values. Therefore, it is purported that aquaponics cannot be achieved simply by positioning a layer of crop onto an aquatic surface; instead, for each factor to be determined, it must be observed constantly and precisely. The assimilation of IoT into the system is crucial as it provides a constant monitoring towards each value crucial to the maximum yield of the two integrated systems: hydroponics and aquaculture. The integration of IoT into aquaponics, however, is still in its infancy with little to no feasible impact as compared to its conventional counterpart, which does not rely on the use of technologies in essence [96].

Previously, agricultural knowledge was passed down through generations within regions, impeding development due to verbal dissemination and slow diffusion across borders. Hence, establishing an ordered manner of knowledge sharing is necessary to benefit the aquaponics community integrating IoT. The very first step in establishing a community for knowledge sharing is the information acquisition. The information first needs to be filtered in order to classify data that is relevant to the community, related to the issues covered, and also true and probable. In doing so, dissemination and differentiation processes allow the community to obtain the final product which is the knowledge.

Knowledge sharing in small-scale aquaponics is crucial for promoting the sustainability and efficiency of this food production system [97]. Despite increasing popularity, there is a lack of proper knowledge and expertise in operating these complex systems, hindering optimal performance and limiting potential benefits. Moreover, knowledge sharing in aquaponics can contribute to addressing issues like rising obesity rates and food deserts by providing communities with access to fresh produce and promoting environmental sustainability. Consequently, promoting knowledge-sharing initiatives in small-scale aquaponics not only enhances the productivity and effectiveness of these systems but also aligns with broader efforts to create a more sustainable and equitable food system.

To foster innovation and sustainability in small-scale aquaponics, it is imperative to address key challenges that hinder progress in this field. As highlighted in the literature [98], aquaponics possesses significant benefits in terms of social, economic, and environmental aspects. However, in order to optimize these advantages, it is crucial to involve all stakeholders, from researchers to decision-makers and consumers. By engaging with the relevant parties, a collaborative framework can be established to promote the commercial development of aquaponics. Additionally, incorporating participatory approaches [99] can enhance success and sustainability by addressing asymmetries in information sharing, promoting reciprocal communication, empowering beneficiaries, increasing involvement in decision-making, and fostering a sense of ownership.

The integration of IoT facilitates real-time data sharing among the broad community of aquaponics through sensors and other communication technologies embedded in various interconnected devices. The collection of data from these devices over the internet and transmission of information assures the synchronous exchange of information in real-time [100]. In terms of knowledge management for the aquaponics community, such devices can measure the quality of water parameters, fish behavior, plant growth, and the particular environmental condition, which is relative to the actual system performance. For example, the sensors incorporated into aquaponics systems could be used for the detection of changes in pH levels, concentrations of ammonia, or water temperature, and to notify them of a central database or other practitioners in aquaponics immediately. This hence paves the way for the real-time monitoring and analysis of the situation toward decision-making and allowing one to draw reactions as far as the occurrence of a potential problem is concerned [100].

## 5. Conclusions

Aquaponics play a pivotal role in addressing the mounting challenges of food demand, highlighting its eco-friendly and sustainable nature. Integrating automation, smart strategies, and IoT technologies into aquaponics systems, such as real-time monitoring, the automatic adjustment of parameters, and computer vision technology for analyzing fish movement behavior, can enhance efficiency and profitability. A small-scale aquaponics system presents a viable and sustainable solution for household food production. A simple aquaponics setup, consisting of a fish tank, a growing medium for plants, and a water pump to circulate the nutrient-rich water, can be easily implemented in a backyard or indoor setting. These compact systems not only provide fresh fish and vegetables but also promote environmental sustainability by minimizing water wastage and reducing the need for chemical fertilizers. To further enhance the economic feasibility and eco-friendliness of these setups, the implementation of solar power can significantly reduce the energy costs associated with running the water pump and grow lights.

One of the major concerns identified in aquaponic farming is the potential for the system to become a breeding ground for mosquitoes. In aquaponics systems, the presence of standing water in fish tanks, grow beds, and other components can inadvertently create suitable habitats for mosquito breeding if proper preventive measures are not taken. This issue becomes more pronounced in hot and humid regions, where the warm temperatures accelerate the mosquito life cycle, leading to rapid population growth. Therefore, it is crucial to think ahead and implement proactive measures by integrating IoT technology that helps detect and prevent mosquito breeding right from the initial setup stage. The future of small-scale aquaponics is bright, but continued research and development are needed. Optimizing system designs, automation, and leveraging technologies like IoT and AI can improve resource management and yields. Additionally, exploring renewable energy sources can enhance sustainability and cost-effectiveness. However, overcoming challenges like nutrient utilization, disease prevention, and public perception is crucial. Establishing best practices and fostering knowledge-sharing communities can address these while policy support can facilitate wider adoption. Integrating aquaponics into urban green infrastructure can further promote its role in sustainable cities.

To further strengthen the investigation into the effectiveness of IoT solutions in diverse aquaponics setups, several specific research questions and experimental designs can be proposed. A research question could focus on the impact of IoT-enabled precision feeding systems on feed conversion ratios and water quality across different fish species. These could be designed as an experiment where multiple aquaponics systems, but with different fish species or IoT-based feeding systems, are run against control systems that run based on traditional methods. Additionally, one could set up a long-term study on the cost-to-benefit ratio coverage by a fully integrated IoT solution in small-scale aquaponics systems by comparing energy consumption to reductions in labor along with increases in productivity.

## Figures and Tables

**Table 1:** Comparison of small-scale and commercial aquaponics.

Characteristics	Small-Scale	Commercial
Size	Few to hundred liters fish tank	Several thousand liters fish tank, with several acres
Location	Indoors or backyards	Large greenhouse
Investment	Relatively low	Require high capital investment
Expertise	Easier to manage with less skills	Skilled workers required
Market	Personal consumption/Locally	Larger markets
IoT technology	Basic	Advanced
Variety	Diverse range of crops and fish species in small quantity	Specific high-value crops and fish species for market demand

## Data Availability

Not applicable.

## Appendix A

**Table A1:** Parameter range for small-scale aquaponics, suitable parameter range for each component of the system, sensors required for parameter detection, and budget.

Parameter	Suggested Range	Aquaculture Range	Hydroponic Range	Nitrification	High Level Effect	Low Level Effect	Sensor	Budget USD	References
pH	6.0–8.0	7.5–8.5	5.5–7.0	7.0–8.5	Disrupt the fish’s osmoregulation; reduced nutrient uptake by plant	Decrease the oxygen-holding capacity of water; growth of harmful bacteria	Multifunctional sensor (pH, light, moisture, TDS, etc.)	$2.10–$5.30	[101,102,103,104]
Water temperature	21 °C–29 °C	25 °C–32 °C	18 °C–30 °C	14 °C–34 °C	Heat stress; lower dissolved oxygen	Less active fish with poor feed conversion; reduced photosynthesis in plants	Water quality detector 5 in 1 (pH/EC/TDS/Salinity/Temperature)	$6.50–$13.20	[77,105,106]
Air temperature	18 °C–30 °C	15 °C–40 °C	25 °C	20 °C–30 °C	Oxygen demand of fish increases; plant prematurely flowers	Slow down the fish’s metabolism and rate of plant growth	Thermistor	$0.21–$2.10	[101,107,108]
Dissolved oxygen	5.0–9.0 mg/L	6.5–8.5 mg/L	5.5–6.5 mg/L	7.0–9.0 mg/L	Gas Bubble Disease in fish	Fish suffocate and die; anaerobic bacteria multiply	Dissolved oxygen meter	$73.40–$83.00	[109]
Relative humidity	50–80%	52–87%	50–60%	50–80%	Fungal diseases on plants	High transpiration causes water loss	Hygrometer	$7.99–$54.99	[110,111,112]
CO_2_ concentration	<20 ppm	<10 ppm	1000–2000 ppm	–	Fish become lethargic due to insufficient oxygen; plants need more micronutrients	–	CO_2_ sensor	$69.99–$75.99	[113,114,115]
Light intensity	300–500 lx	20–40 lx	300–400 lx	350 lx	Increase cortisol levels in fish; higher anthocyanin and sugar in plants	Lower thyroid hormone levels; reduced shoot and root growth	Multifunctional sensor (pH, light, moisture, TDS, etc.)	$2.10–$5.30	[20,105,116,117]
Illumination duration	12 h	10–14 h	12 h	10–16 h	Scorched and pale leaves	Decreases total biomass and dry weight	–	–	[20,118,119,120]
Flow speed	0.8 L/min–8.0 L/min	1.0 L/min–1.5 L/min	2 L/min–4 L/min	–	Energy intensive; less nutrient contact for roots	Inadequate nutrient distribution; buildup of waste materials	Flow meter	$11.99–$29.99	[103,121,122,123]
Water hardness	50–150 ppm	50–150 ppm	100–150 ppm	–	Reduced oxygen exchange in root zone	Lack of micronutrients for plant growth	Water hardness test kit	$8.99–$17.99	[113,124]
Total dissolved solid	400–700 ppm	400–600 ppm	400–500 ppm	750–1500 ppm	Increase water temperature; decrease photosynthesis	Lack some essential plant micronutrients	Multifunctional sensor (pH, light, moisture, TDS, etc.)	$2.10–$5.30	[64,125,126]
Nitrites	<1 mg/L	<1 mg/L	<5 mg/L	–	Induce stress; impairs oxygen uptake	–	7 in 1 Aquarium Test Strips	$8.99–$19.99	[66,127,128]
Nitrates	100–200 mg/L	200–500 mg/L	15–1000 mg/L	–	Algal blooms	Nutrient deficiency for plants	7 in 1 Aquarium Test Strips	$8.99–$19.99	[129,130,131,132]
